# Heterogeneity and spatiotemporal evolution characteristics of regional emergency logistics response capacity: a case of China

**DOI:** 10.3389/fpubh.2025.1461354

**Published:** 2025-03-06

**Authors:** Heng Chen, Xianglong Lin, Yuan Guo, Xianchao Qi

**Affiliations:** School of Management, Xi’an Polytechnic University, Xi’an, China

**Keywords:** emergency logistics response capacity, regional heterogeneity, spatiotemporal evolution characteristics, entropy-weighted TOPSIS method, Markov chain

## Abstract

Public emergencies have surged worldwide, highlighting the critical role of emergency logistics in disaster relief. This study evaluates the heterogeneity and spatiotemporal evolution characteristics of regional emergency logistics response capacity in China using the entropy-weighted TOPSIS method, Dagum Gini coefficient, kernel density estimation, and Markov chain analysis. The emergency logistics response capacities of 30 provinces and four major economic regions (Eastern, Central, Western, Northeastern) were assessed from 2012 to 2021. Key findings reveal: (1) Provincial emergency logistics response capacities improved significantly nationwide, yet regional imbalances remain pronounced. (2) Regional heterogeneity expanded, driven primarily by inter-regional disparities. (3) Temporal analysis shows steady growth across all economic regions without polarization, with the Eastern region achieving the highest mean capacity and growth rate. (4) Spatial evolution demonstrates continuity, as Markov chain analysis reveals gradual transitions between adjacent capacity levels but limited leapfrog development. Spatial factors exert dual effects: proximity to high-capacity regions facilitates upgrades for moderate-level areas, yet suppresses low capacity regions. These findings emphasize persistent structural gaps in infrastructure, resource allocation, and governance.

## 1 Introduction

In recent years, there has been an observable trend toward an increased intensity and frequency of natural disasters and other sudden public emergencies, indicating a new normal (1). China, being one of the countries most severely affected by natural disasters, is characterized by a diverse array of disaster types, a wide geographic distribution, frequent occurrences, and substantial losses (2). The “2021 Global Natural Disaster Assessment Report” highlights that China was struck by 21 significant natural disasters in 2021 alone, affecting 107 million people and leading to 765 fatalities and 102 missing persons, with direct economic losses amounting to an alarming 334.02 billion yuan (46.15 billion dollar). These public emergencies not only result in severe human casualties and financial losses but also pose formidable challenges to the disaster management and emergency response systems (3). Within this context, emergency logistics emerges as a pivotal element, providing essential support for the supply demands and personnel deployment during such crises (4). The effectiveness of emergency logistics is crucial for mitigating disaster impacts and ensuring the prompt delivery of necessary goods and services to the affected regions (5). Accordingly, this aspect of emergency management has attracted considerable attention from academic circles, governmental bodies, and the public at large.

Numerous scholars have investigated the assessment of emergency logistics response capacity employing methodologies such as the Analytic Hierarchy Process (AHP) and Fuzzy Comprehensive Evaluation (FCE) (6–9). However, these existing methodologies exhibit limitations due to the subjectivity and randomness in selecting indicator weights, compromising the scientific validity and reliability of the evaluations. Despite these challenges, some researchers have applied case study methods to delve into emergency logistics in specific contexts (10–12), focusing predominantly on particular disasters or locales from a micro perspective, thus limiting broader regional evaluations of emergency logistics.

Additionally, the comprehensive nature of emergency logistics response capacity (Hereinafter referred to as ELRC) remains underexplored in the literature, further complicated by significant regional disparities in China’s logistics development (13), which have led to evident regional heterogeneity in ELRC. Recent initiatives in China have included the introduction of coordinated regional logistics development plans, which have significantly influenced the landscape of regional logistics development of regional logistics development patterns. However, existing research has yet to adequately address the dynamic characteristics of ELRC, thus leaving a gap in the literature regarding its evolving nature.

The objectives of this study are threefold: First, it aims to develop a comprehensive indicator system to evaluate ELRC, enabling the scientific and objective measurement across different regions in China. Second, the study analyzes regional heterogeneities in China’s ELRC. Third, it investigates the spatiotemporal evolution of these capacities. This research offers a novel perspective and theoretical foundation, enhancing the understanding of China’s regional ELRC. The findings are intended to provide valuable insights for crafting more scientifically robust and rational emergency logistics policies. This study adopts a regional perspective, broadening the scope of existing literature by providing an in-depth analysis of the factors constituting ELRC and establishing an integrative index system. The entropy-weighted TOPSIS method is employed for measurement, enhancing the rigor and credibility of the results. The use of the Dagum Gini coefficient for heterogeneity analysis introduces a novel approach for understanding inter-regional disparities. Furthermore, the spatiotemporal evolution analysis enhances the understanding of the changing patterns and characteristics of emergency logistics. The paper is organized as follows: Section 2 provides a literature review. Section 3 describes the research methodology and data sources. Section 4 outlines the components and measurement results of ELRC. Section 5 discusses the heterogeneity and origins of regional differences in ELRC within China. Section 6 examines the spatiotemporal evolution of these capacities. Section 7 discusses the study’s novelty, significance, limitations, and directions for future research. Section 8 concludes with the study’s implications and recommendations for policy.

## 2 Literature review

In the field of emergency logistics, contemporary research has primarily focused on three distinct areas: the concept and connotation, the various patterns and models, and the methods of evaluation.

### 2.1 Concept and connotation of emergency logistics

Emergency logistics, internationally recognized as a well-established discipline, is defined as the urgent logistical activities designed to ensure efficient transportation and allocation of relief materials, equipment, and personnel in response to unforeseen events such as public health emergencies and natural disasters. Its core objective lies in delivering critical resources to disaster-stricken areas within the shortest possible timeframe to minimize losses and preserve lives (14). Key characteristics of this field include its unpredictability and exceptionality (15, 16), stochastic demand and selectivity (17, 18), uneven flow distribution (19, 20), time-critical constraints, and the imperative to prioritize social welfare (21). Due to the inherent unpredictability of such events, emergency logistics frequently encounters challenges stemming from the scarcity of historical planning data (22). Nevertheless, it has evolved into an indispensable component of disaster mitigation strategies, playing a pivotal role in managing crises effectively and delivering humanitarian aid (23).

Distinct from conventional social logistics, which aims to sustain societal operations and enhance quality of life (24), emergency logistics prioritizes time-efficiency optimization and loss minimization (25). Despite this divergence, scholars have identified shared foundational components between the two systems, including carriers, flow direction, flow, and flow rate (14). Consequently, evaluations of ELRC must be grounded in these intrinsic characteristics. Given the resource scarcity inherent to emergency logistics and its frequent deviation from full decision-maker control (26), a robust material foundation—encompassing infrastructure, transportation assets, and skilled personnel—is critical to ensure timely support for affected populations through coordinated material, human, informational, and service flows (27, 28).

Functionally, emergency logistics encompasses three primary tasks: (1) delivering relief supplies, (2) evacuating and relocating affected populations, and (3) transporting casualties to medical facilities to ensure safety in disaster zones (29). This necessitates enhanced resource allocation capabilities, operationalized through metrics such as freight volume and turnover rates, to guarantee both scale and efficiency in material distribution. Simultaneously, logistics velocity and operational efficacy significantly influence overall responsiveness. First, response capacity correlates closely with roadway hierarchy and transportation network density, which determine flow velocity and directly impact timeliness. Second, the ratio of logistics output to resource input serves as a critical indicator of utilization efficiency, reflecting the operational effectiveness of response systems—a particularly vital consideration in resource-constrained emergency contexts.

It is noteworthy that while transportation metrics such as freight volume and road network density are conventionally employed to gauge industrial development levels, extant research demonstrates their critical role in reflecting system redundancy and response resilience within emergency logistics contexts (27). For instance, Sheu (27) empirically validated through case studies that the spatial density of road freight volume exhibits a significant positive correlation with disaster relief allocation efficiency. Further building on this, Zhang et al. (30) concluded that the proportion of high-grade roadway has a direct impact on supply chain reliability under extreme conditions, and thus the proportion of high-grade roadway can be identified as a key predictor of emergency logistics timeliness. Thus, this study contends that transportation metrics in ELRC assessments transcend mere industrial scale representation, instead deriving their analytical value from context-specific explanatory power in crisis scenarios.

Beyond these foundational components, emergency logistics—serving as a pivotal support framework and critical safeguard mechanism for public emergencies—necessitates integration with real-world contextual factors, particularly governmental coordination mechanisms and digitalization initiatives, to address the complexities inherent in contemporary crisis management.

### 2.2 Patterns and models of emergency logistics

In the domain of emergency logistics, researchers have innovatively developed various efficient logistics and resource allocation patterns by incorporating advanced technologies such as cloud platforms, artificial intelligence, and blockchain (31–33). Concurrently, Banomyong and Sopadang enhanced the emergency logistics response model by applying the Monte Carlo simulation technique (10). Similarly, Liu et al. (34) developed data-driven models for optimizing emergency logistics networks, while Wang et al. (35) and Chen et al. (36) utilized simulation techniques to evaluate and enhance the reliability and design of these networks.

Further expanding the scope of this research, scholars both domestically and internationally have devoted considerable effort to refining models for emergency logistics facility location selection (37–40), transportation route planning for rescue materials (41–44), and integrated location-path planning (45–47). In addition, models focusing on emergency logistics decision planning (47–49) and regional emergency dispatch (50, 51) have also received significant scholarly attention and discussion.

### 2.3 Evaluation of emergency logistics

In the field of emergency logistics evaluation research, scholars have extensively explored aspects related to reliability, performance, and capability. Initially, Chen et al. (6) introduced the concept of emergency logistics supply chain reliability and evaluated it using the Analytic Hierarchy Process (AHP). Subsequently, Gong et al. (52) conducted a case study to assess the reliability of the emergency logistics system, employing the Fuzzy Entropy Clustering Method for their evaluation. Xu et al. (7) further developed an emergency supply chain reliability evaluation model incorporating assurance mechanisms and information systems, utilizing the AHP and Entropy Weight Method (EWM). They validated the effectiveness and applicability of this model through practical examples. Additionally, Zhang et al. (53) explored the essence of urban emergency logistics system reliability, integrating practical cases and applying the Fuzzy Comprehensive Evaluation (FCE) to assess its reliability.

Regarding performance evaluation, Liu et al. (54) utilized the fuzzy entropy-weighted TOPSIS method with Multi-Granularity Linguistic Assessment^1^ (MGLA) information to assess emergency logistics performance in five severely affected areas during the Wenchuan earthquake. Ji and Zhang (55) proposed a performance evaluation method based on the extensible matter-element model, which resolved incompatibility and contradictions among evaluation indicators, ensuring objectivity and scientificity in performance assessments. Zhang et al. (56) developed an evidence-based emergency logistics performance evaluation model using a specific evaluation method, validated through an instance involving the distribution of emergency resources for flood control along the Jingjiang River. Li et al. (57) adopted multiple methods, including the Delphi Method, AHP, Grey System Theory, and Fuzzy Evaluation, to achieve the most rational performance evaluation results at different stages of the emergency logistics system.

In the domain of capability evaluation, Deng et al. (9) constructed an evaluation index system for ELRC, assessing it using the Fuzzy Grey Comprehensive Evaluation method. Zhang et al. (58) established a dynamic evaluation model of emergency logistics capability based on the evaluation index system for COVID-19 and combined it with a BP neural network. Yang et al. (59) adopted a multi-attribute decision-making technique grounded in probabilistic linguistic terminology for the evaluation of ELRC. Lin et al. (8) developed an evaluation index system for natural disaster emergency logistics capabilities and used the Analytic Network Process (ANP) and multi-level grey evaluation method for the assessment. Sun et al. (60) applied the Fuzzy-AHP to evaluate the ELRC during the Wenchuan, Qinghai, and Lushan earthquakes. Chen and Ke (61) effectively evaluated emergency capabilities comprehensively for five emergency logistics decision-making units using the FCE method based on data envelopment analysis.

Upon systematically summarizing existing research achievements, it is apparent that both domestic and international scholars have explored the concept and connotation of emergency logistics, leading to the establishment of a well-defined theoretical structure and research system. However, the existing relevant research results predominantly focus on qualitative research and have not been further applied to quantitative analysis of ELRC. Regarding research on emergency logistics patterns and model optimization, many studies approach the topic from the mathematical model construction or simulation perspective, focusing mainly on micro-level emergency logistics decision-making and scheduling aspects. Nevertheless, there is a lack of emphasis on identifying regional ELRC. The ultimate objective of both emergency logistics decision-making and scheduling is to enhance ELRC. However, without effectively discerning the current state and spatiotemporal evolution characteristics of ELRC in different regions, both site selection and decision-making in emergency logistics would eventually lack a solid foundation.

Currently, in emergency logistics evaluation research, scholars have discussed various perspectives, such as reliability, performance, and capabilities. However, the existing evaluation methods chosen for study often lean toward subjective approaches, such as the AHP and FCE. Relying on subjective factors to select evaluation indicators may lead to potential inadequacies and a lack of comprehensiveness in the design of evaluation systems. Consequently, the scientificity and accuracy of evaluation results may be undermined. Furthermore, most studies adopt case study methods, focusing primarily on specific locations or particular natural disasters from a micro-level perspective, lacking exploration of a macro-regional view. Moreover, emergency logistics is a complex and multifaceted system, and ELRC is a comprehensive capability. The existing research frequently fails to reflect all the constituent factors involved fully.

Based on existing domestic and international research, this study conducts several extensions. Firstly, it analyzes the constituent factors contributing to ELRC and develops a comprehensive index system. Then, the index system is utilized with the entropy-weighted TOPSIS method to measure the ELRC of 30 provincial administrative regions and four major economic regions in China from 2012 to 2021. Secondly, the study explores the regional heterogeneity of China’s ELRC and its sources using the Dagum Gini coefficient. Thirdly, the study utilizes kernel density estimation and Markov chain analysis methods to examine the spatiotemporal evolution process and characteristics of China’s regional ELRC.

## 3 Research methods and data

### 3.1 Research methods

#### 3.1.1 Entropy-weighted TOPSIS method

In prior research on logistics evaluation, the Multi-Criteria Decision Making (MCDM) method has been extensively employed to address decision-making scenarios involving multiple criteria or attributes. Notable MCDM techniques such as the Analytic Hierarchy Process (AHP) (62), Technique for Order Preference by Similarity to Ideal Solution (TOPSIS) (63, 64), and Multi-objective Linear Programming (65) have been frequently utilized. However, the AHP often involves complex calculations and its criteria selection can introduce subjectivity. Conversely, Multi-objective Linear Programming is limited to linear problem contexts. TOPSIS, which evaluates options by considering both the negative-ideal and ideal solutions, offers a comprehensive and intuitive assessment, thus its widespread adoption. Nonetheless, the method’s reliance on subjective judgment when establishing weights could potentially impact the final decision outcomes.

In response to these limitations, the Entropy Weight Method, which derives weights from the inherent variability of the data, provides a more objective alternative. The entropy-weighted TOPSIS (Technique for Order Preference by Similarity to Ideal Solution) method combines the entropy weight method and TOPSIS to objectively evaluate multidimensional decision-making problems. First, entropy weight method calculates indicator weights rooted in data dispersion, minimizing subjective bias by assigning higher weights to indicators with greater information utility. Subsequently, TOPSIS ranks alternatives by measuring their Euclidean distances to the negative and positive ideal solutions, ensuring a comprehensive assessment of relative performance. This approach offers three key advantages: (1) objectivity in weight determination through data-driven entropy values, (2) robustness in handling heterogeneous or conflicting criteria, and (3) interpretability via clear proximity scores (0–1), which quantify the gap between observed and optimal performance. In this study, it effectively captures the complexity of emergency logistics systems while avoiding arbitrary assumptions in traditional methods (e.g., AHP). This research therefore adopts the entropy-weighted TOPSIS for evaluation purposes. The initial step involves assigning weights to various measurement indicators using the Entropy Weight Method. Subsequently, TOPSIS is applied to quantitatively rank the ELRC of different provinces. The specific calculation steps are outlined as follows:

1.  Standardized processing of raw data

In order to compare indicators across various provinces and years, this study utilized the Min-Max Scaling technique to normalize the data, thereby removing its dimensionality.

2.  Weight calculation

Based on the entropy weighting method, this paper establishes the following weighting equations (Equations 1–3):


(1) $$p_{\theta ij}=\frac{{x^{\prime }}_{\theta ij}}{\Sigma_{\theta =1}^{r}\Sigma_{i=1}^{n}{x^{\prime }}_{\theta ij}}$$

(2) $$e_{j}=-\frac{\Sigma_{\theta =1}^{r}\Sigma_{i=1}^{n}\left(p_{\theta ij}^{*}\ln p_{\theta ij}\right)}{\ln rn}$$

(3) $$w_{j}=\frac{1-e_{j}}{\Sigma_{j=1}^{n}\left(1-e_{j}\right)}$$

Among these, there are *r* years, *n* provinces, and *m* indicators, $x_{\theta ij}$ Represents the value of the *j* indicator for the *i* province in the θ year. Here, θ=1,2,…,r;i=1,2,…,n;j=1,2…,m . $p_{\theta ij}$ represents the characteristic proportion value of the *j* indicator for the *i* province. $e_{j}$ denotes the information entropy of the *j* indicator. *w_j_* signifies the weight of the *j* indicator, $w_{j}\in \left[0,1\right]$ and $\overset{n}{\underset{j=1}{\Sigma }}w_{j}=1$ .

3.  Create the weighted matrix *Z*

(4) $$z_{\theta ij}=w_{j}\times x_{\prime }^{\theta ij}\left(\theta =1,\;2,\ldots ,r,\;i=1,\;2,\;n;\;j=1,\;2,\ldots ,\;m\right)$$

(5) $$Z={\left(z_{\theta ij}\right)}_{r\times m\times n}$$

In Equations 4, 5, $z_{\theta ij}$ represents the weighted decision score, and *Z* is the weighted decision matrix composed of all weighted decision scores.

4.  Calculate Euclidean distance

(6) $$d_{j}^{+}=\max(z_{\theta ij}),\;\;D_{\theta i}^{+}=\sqrt{\overset{n}{\underset{j=1}{\Sigma }}{\left(d_{j}^{+}-z_{\theta \mathrm{ij}}\right)}^{2}}$$

(7) $$d_{j}^{-}=\min\left(z_{\theta ij}\right),\;\;D_{\theta i}^{-}=\sqrt{\overset{n}{\underset{j=1}{\Sigma }}{\left(d_{j}^{-}-z_{\theta ij}\right)}^{2}}$$

In Equations 6, 7, $d_{j}^{+}$ and $d_{j}^{-}$ respectively represent the positive and negative ideal solutions. $D_{\theta i}^{+}$ and $D_{\theta i}^{-}$ represent the Euclidean distances between the actual level of ELRC for each province and the positive and negative ideal solutions, respectively.

5.  Calculate the ELRC $C_{\theta i}$ for each province:

(8) $$C_{\theta i}=\frac{D_{\theta i}^{-}}{D_{\theta i}^{+}+D_{\theta i}^{-}}$$

In Equation 8, $C_{\theta i}\in \left[0,\;\;1\right]$ . The closer the value of $C_{\theta i}$ is to 1, the higher the ELRC of that region.

#### 3.1.2 Dagum Gini coefficient

The Dagum Gini coefficient differs from the traditional Gini coefficient, coefficient of variation, and Theil index by factoring in the distribution of sub-samples. It considers issues like cross-overlap between samples (66). Consequently, the Dagum Gin coefficient effectively addresses the source of regional disparities, making it highly advantageous in analyzing spatial imbalances (67). It is commonly used to describe the issue of regional development imbalance, particularly in research related to disparities in residents’ income and economic development across regions (58, 68). Additionally, in this study, it serves as a crucial tool to explore the heterogeneity of ELRC across different regions in China. The formula for calculating the Dagum Gini coefficient is shown in Equation 9:


(9) $$G=\frac{\overset{k}{\underset{j=1}{\Sigma }}\overset{k}{\underset{h=1}{\Sigma }}\overset{n_{j}}{\underset{i=1}{\Sigma }}\overset{n_{h}}{\underset{r=1}{\Sigma }}\left|y_{ji}-y_{hr}\right|}{2\mu n^{2}}$$

Among these, *G* represents the overall Gini coefficient, *n* is the number of provinces, and *k* is the number of regions. *i* and *r* represent the number of provinces within each region. *n_j_* (*n_h_*) represents the number of provinces in the *j* (*h*) region. *y_ji_* (*y_hr_*) represents the ELRC value of the *i* (*r*) province in the *j* (*h*) region. *μ* represents the mean of ELRC for each province. Additionally, the Dagum Gini coefficient, using the subgroup decomposition method, allows for further breakdown of the overall Gini coefficient (*G*) into three distinct components: hypervariance density (*G_t_*), inter-regional heterogeneity (*G_nb_*), and intra-regional heterogeneity (*G_w_*) (Equation 10):


(10) $$G=G_{w}+G_{nb}+G_{t}$$

#### 3.1.3 Kernel density estimation

Kernel density estimation is a widely used non-parametric method for studying the uneven distribution of samples (69). It enables the estimation of the probability density of random variables and describes the dynamic evolution trend of their distribution through continuous density curves. In this study, kernel density estimation is used to reveal the dynamic evolutionary trends of regional ELRC in China under the time dimension.

In this research, the density function of regional ELRC is represented by *f* (*x*). And the probability density estimate at point *x* is shown in Equation 11:


(11) $$f\left(x\right)=\frac{1}{Nn}\overset{n}{\underset{i=1}{\Sigma }}k\left(\frac{x_{i}-x)}{h}\right)$$

In the above equation, *h* represents the bandwidth, *N* denotes the number of observed values, and k(•) represents the kernel density function. *x_i_* represents independently and identically distributed observed values, while *x* represents the mean. This study selects the most commonly used Gaussian kernel function to investigate the dynamic evolution of China’s regional ELRC distribution. The expression is shown in Equation 12:


(12) $$K\left(x\right)=\frac{1}{\sqrt{2\pi }}\exp\left[-\frac{x^{2}}{2}\right]$$

#### 3.1.4 Markov chain

The Markov chain is a stochastic process {X(t),t∈T} , where its index set *T* corresponds to different periods, and the finite types correspond to the number of types of random variables. Therefore, for all periods *t* and all types *i* and *j*, the Equation 13 should hold:


(13) $$\begin{array}{l} P\left\{X\left(t+1\right)=j|X\left(t\right)=i,\;X\left(t-1\right)=i_{t-i},\;\Lambda ,\;X\left(0\right)=i_{0}\right\}\\ =P\left\{X\left(t+1\right)=j|X\left(t\right)=i\right\} \end{array}$$

Indeed, the Markov chain is commonly utilized for discretizing continuous attribute values of geographical phenomena in different periods. And, it can effectively avoid the impact of data non-temporality on prediction accuracy (70). Thus, in this research, the construction of a Markov chain is used to explore the probabilities and rules of relative transitions in China’s regional ELRC. The Markov chain typically uses data categorization to divide it into *k* different types. The distribution of a certain type at time *t* is represented using a 1 × k state probability vector $E_{t}=\left[E_{1,t},E_{2,t},\ldots ,E_{k,t}\right]$ . The entire process of state transition of the object can be represented using a Markov probability transition matrix of size *k × k*, with probability value *M_ij_*. *M_ij_* denotes the probability value of a spatial unit of type *i* at time *t* transitioning to type *j* at time *t* + 1. The specific formula is shown in Equation 14:


(14) $$M_{ij}=n_{ij}/n_{i}$$

Among these, *n_ij_* represents the cumulative count of spatial units transitioning from type *i* at time *t* to type *j* at time *t* + 1. *n_i_* represents the total count of spatial units of type *i* across all time steps during the research period.

### 3.2 Research data

Given the availability and scientific validity of the data, this study focuses on 30 provincial administrative regions, including Beijing, Shaanxi, Henan, Shandong, etc. (excluding the Hong Kong, Tibet Autonomous Region, Taiwan, and Macau).

The regional heterogeneity of ELRC can be categorized from multiple dimensions, such as disaster types, disaster frequency, transportation hub types, emergency evacuation functions, and economic factors. This paper conducts a regional heterogeneity analysis based on the division of China’s four major economic regions for several reasons: First, the ELRC is highly dependent on long-term infrastructure investments and technological upgrades, while economic levels directly impact regional fiscal capacity and resource allocation priorities. Economically developed regions have higher fiscal budgets and can systematically build high-grade highways, among other infrastructure. Second, the division of China’s four economic regions is itself embedded in national strategie, and the allocation of policy resources directly shapes regional emergency capabilities. The Eastern region attracts global logistics companies through free trade zone policies, forming an emergency network of public-private cooperation, while the western region relies on central government transfer payments, with the efficiency of fund use constrained by local governance capabilities. Third, ELRC must address complex disasters (e.g., the COVID-19 pandemic combined with extreme weather), and economic strength determines the region’s system resilience under multiple shocks. A single disaster-oriented classification cannot capture such complex resilience mechanisms. Finally, the division of economic regions is highly compatible with the official statistical system, making it easier for monitoring and assessment. Economic region data is standardized, continuous, and policy-intervenable, which is more conducive to building dynamic evaluation models. In contrast, disaster types or transportation hub classifications often require cross-departmental data integration, which may present inconsistencies in scope and timeliness risks. The advantages and disadvantages of specific classification methods are shown in Table 1. In summary, this paper adopts economic regions as the basis for classification.

**Table 1:** Comparison table of classification methods.

Classification method	Advantages	Limitations (for ELRC research)
Disaster type classification	Reflects regional risk exposure differences	1. Cannot explain capability gaps within the same risk zone; 2. Ignores the decisive role of economic foundation in disaster reduction resources; 3. Dynamic changes in disaster types (such as new risks from climate change), resulting in insufficient classification stability.
Transportation hub classification	Identifies key logistics nodes	1. Ignores the support of the economic hinterland for the hub functions; 2. Difficult to quantify the emergency collaboration potential of non-hub areas; 3. Node importance changes dynamically with supply chain restructuring.
Economic region classification	1. Reveals structural differences in resource allocation; 2. Aligns with policy tools; 3. Captures comprehensive resilience mechanisms.	Needs to combine subgroup analysis (such as disaster types, hub levels) to refine heterogeneity.

**Figure 1:**
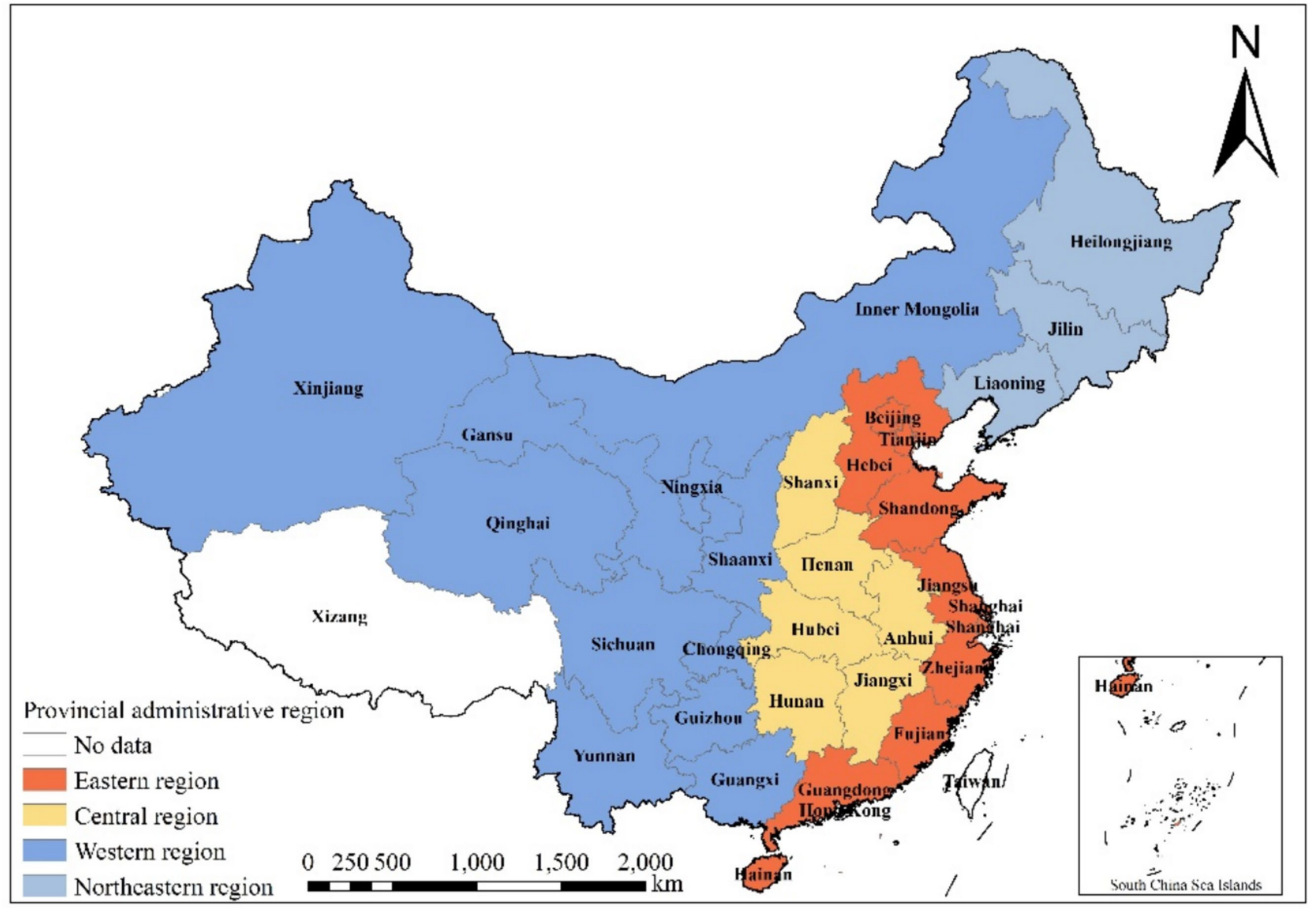
Distribution of four major economic regions in China.

Simultaneously, China categorizes the entirety of its territories into four primary economic regions based on local natural conditions, economic foundations, development levels, and degrees of external openness: Eastern, Central, Western, and Northeastern. This classification aims to foster coordinated regional development. To comprehensively understand the evolving dynamics of regional ELRC, this paper refers to the economic regional classifications provided by the National Bureau of Statistics, as depicted in Figure 1. Besides, 2012 was designated as the baseline year, as it corresponds with the introduction of China’s first policy aimed at developing the emergency industry, titled “Opinions on Accelerating the Development of the Emergency Industry,” issued by the General Office of the State Council. Additionally, in 2018, the Ministry of Emergency Management was established in China, and corresponding emergency management departments were set up in various provinces. Due to the lag in provincial data, it is relatively difficult to obtain data after 2021. Additionally, the period from 2011 to 2021 aligns with China’s 12th and 13th Five-Year Plans, during which emergency logistics policies remained relatively stable. Extending the analysis to 2023 would introduce noise from post-pandemic policy adjustments. Therefore, this study leverages observational metrics systematically documented over a ten-year continuum, commencing in 2012 and concluding in 2021. Drawing from previous research experience, a 10-year period is deemed an appropriate timeframe for analyzing the cyclical fluctuations of a subject, as it captures long-term trends and cycles while ensuring data relevance and timeliness. All original data required for this study are sourced from the website of National Bureau of Statistics^2^ and the respective year’s “China Statistical Yearbook.” And some indicators are derived through calculations based on the original data, an example is the ratio of fixed asset investment in the logistics industry to the overall investment in fixed assets. For the rare cases of missing data, the mean imputation was utilized, whereby missing values were replaced by the average observed value of the respective variable.

## 4 Components and measurement of regional emergency logistics response capacity in China

### 4.1 Components and measurement indicators

Similar to regular logistics, emergency logistics consists of several key components such as flow direction, carriers, flow rate, and flow (71). There is a consensus among scholars who study logistics capacity and, specifically, ELRC, on the measurement indicators for carrier capacity, flow rate, and velocity in logistics systems. Building on these elements, this study adopts the analytical framework for evaluating ELRC proposed by Xi (72), selecting metrics such as emergency logistics flow response capacity, emergency logistics carrier carrying capacity, emergency logistics flow velocity response capacity, regional informatization response capacity, and emergency logistics flow efficiency response capacity to identify the constitutive components of ELRC.

Furthermore, failure to incorporate contextual factors into the metric implementation of regional ELRC risks hindering capacity enhancement. As a pivotal support framework and critical safeguard mechanism for public emergencies, emergency logistics thus necessitates integrating practical considerations—particularly those arising in real-world crisis scenarios—to optimize its operational efficacy. Building on this rationale, this study posits that ensuring the availability of public medical resources during emergencies constitutes a governmental obligation across administrative tiers, while managing and coordinating emergency logistics necessitates multi-tiered governmental coordination (73–76). To operationalize this framework, Li (31) introduced social emergency security capacity as a critical dimension of ELRC. Furthermore, empirical studies identify regional informatization response capacity as a pivotal determinant of adaptation rates in logistical response systems (77). Consequently, societal emergency safeguard capacity and regional digitalization capacity are integrated as core components of ELRC. The specific composition of the indicator system is detailed in Table 2.

**Table 2:** The index system of regional emergency logistics response capacity.

First-level indicators	Second-level indicators	Third-level indicators	Weight coefficient
Emergency logistics carrier carrying capacity	Logistics infrastructure	Transport network density	3.60%
	Logistics transportation equipment ownership	Civil vehicle ownership	3.69%
		Ownership of road-operated vehicles	2.79%
	Logistics carrier capacity	Express business volume	15.90%
	Logistics industry practitioners	Number of employments in railway, road, aviation, handing and other transportation, warehousing, and postal services	2.33%
Emergency logistics flow response capacity	Freight volume	Road freight volume	3.03%
		Railway freight volume	8.29%
	Freight turnover	Railway freight turnover	8.16%
		Road freight turnover	5.02%
Emergency logistics flow velocity response capacity	Proportion of classified roads	Proportion of expressways	0.77%
		Proportion of first-class roads	4.27%
		Proportion of second-class roads	1.28%
Emergency logistics flow efficiency response capacity	Completed logistics volume per unit manpower	Ratio of freight volume to employed personnel	2.59%
		Ratio of freight turnover to employed personnel	1.91%
	Completed logistics volume per unit capital investment	Ratio of freight volume to logistics industry capital investment	3.18%
		Freight turnover relative to logistics industry capital investment	6.80%
Regional informatization response capacity	Telecommunication business volume	Total postal and telecommunications business volume	8.20%
	Network infrastructure	Optical fiber line length	3.62%
	Regional communication level	Number of mobile phone users	3.01%
		Number of internet broadband access ports	3.78%
Social emergency security capacity	Rescue support foundation	Number of beds in health institutions	2.60%
		Number of health technicians	2.51%
		Number of disease prevention and control centers	1.90%
	Regional policy support	Proportion of local fiscal transportation expenditure in total expenditure	0.78%

### 4.2 Analysis of measurement results

Based on the indicator system mentioned above, this study employed Stata17 software and applied the entropy-weighted TOPSIS method to calculate the ELRC of 30 provincial administrative regions in China from 2012 to 2021. And the results are presented in Table 3. Firstly, upon comparing the measurement values of each province during the period 2012–2021, a significant improvement in the ELRC is evident across all provinces in China. Secondly, from the provincial average values, it is clear that Qinghai Province exhibits the weakest ELRC, with an average value of only 0.0552. Conversely, Guangdong Province demonstrates the strongest ELRC, with a notably high average value of 0.4696. This indicates a significant disparity in ELRC among the various provinces in China. Finally, upon observing the numerical distribution of ELRC, it is evident that provinces with stronger ELRC are primarily concentrated in the Eastern region. However, provinces in the Western region display relatively weaker ELRC. This observation highlights the clear regional imbalance in China’s ELRC.

**Table 3:** Calculation results of emergency logistics response capacities of provinces in China.

Province	2012	2013	2014	2015	2016	2017	2018	2019	2020	2021	Mean
Beijing	0.1938	0.2029	0.2033	0.2029	0.2126	0.2186	0.2252	0.2280	0.2451	0.2525	0.2185
Hebei	0.2388	0.2533	0.2656	0.2637	0.2758	0.2981	0.3211	0.3317	0.3542	0.3722	0.2975
Tianjin	0.1425	0.1299	0.1333	0.1282	0.1301	0.1340	0.1368	0.1418	0.1433	0.1435	0.1363
Shanxi	0.1793	0.1882	0.1967	0.1922	0.1941	0.2322	0.2462	0.2518	0.2579	0.2703	0.2209
Inner Mongolia	0.1908	0.1835	0.1878	0.1791	0.1834	0.2108	0.2330	0.2216	0.2180	0.2222	0.2030
Liaoning	0.2176	0.2210	0.2249	0.2258	0.2489	0.2614	0.2815	0.2705	0.2579	0.2637	0.2473
Jilin	0.1017	0.0982	0.0985	0.0951	0.0979	0.1051	0.1101	0.1128	0.1197	0.1222	0.1061
Heilongjiang	0.1348	0.1383	0.1363	0.1321	0.1359	0.1441	0.1430	0.1481	0.1465	0.1481	0.1407
Shanghai	0.2206	0.2406	0.2663	0.2540	0.2570	0.2807	0.2916	0.3220	0.3406	0.3600	0.2833
Jiangsu	0.2158	0.2487	0.2612	0.2730	0.2840	0.3066	0.3320	0.3686	0.3960	0.4274	0.3113
Zhejiang	0.1893	0.1991	0.2145	0.2348	0.2498	0.2740	0.3011	0.3354	0.3800	0.4147	0.2793
Fujian	0.1194	0.1358	0.1403	0.1470	0.1497	0.1643	0.1806	0.1892	0.1971	0.2100	0.1633
Anhui	0.2178	0.2223	0.2271	0.1980	0.2025	0.2169	0.2354	0.2270	0.2380	0.2412	0.2226
Jiangxi	0.1269	0.1359	0.1361	0.1326	0.1362	0.1561	0.1670	0.1567	0.1655	0.1714	0.1484
Shandong	0.2811	0.2795	0.2837	0.2898	0.3110	0.3250	0.3509	0.3686	0.3958	0.4133	0.3299
Henan	0.2895	0.2725	0.2858	0.2879	0.3017	0.3174	0.3380	0.3433	0.3634	0.3741	0.3174
Hubei	0.1306	0.1482	0.1566	0.1654	0.1709	0.1803	0.1939	0.1926	0.1929	0.2027	0.1734
Hunan	0.1472	0.1497	0.1576	0.1591	0.1650	0.1783	0.1901	0.1879	0.2010	0.2091	0.1745
Guangxi	0.1346	0.1397	0.1438	0.1445	0.1490	0.1609	0.1741	0.1764	0.1932	0.2022	0.1618
Guangdong	0.2887	0.3699	0.3875	0.4058	0.4310	0.4755	0.5357	0.5531	0.5954	0.6532	0.4696
Hainan	0.0683	0.0444	0.0538	0.0531	0.0516	0.0508	0.0536	0.0687	0.0855	0.0896	0.0619
Chongqing	0.0799	0.0897	0.0926	0.0960	0.0989	0.1067	0.1123	0.1141	0.1181	0.1241	0.1032
Sichuan	0.1555	0.1866	0.1964	0.2035	0.2119	0.2229	0.2486	0.2513	0.2672	0.2863	0.2230
Guizhou	0.0759	0.0849	0.0915	0.0944	0.0978	0.1045	0.1160	0.1168	0.1228	0.1305	0.1035
Yunnan	0.0993	0.1223	0.1234	0.1270	0.1305	0.1402	0.1476	0.1530	0.1612	0.1716	0.1376
Shaanxi	0.1413	0.1531	0.1656	0.1621	0.1700	0.1801	0.1901	0.1934	0.2056	0.2156	0.1777
Gansu	0.0892	0.0931	0.0931	0.0927	0.0944	0.1014	0.1120	0.1113	0.1127	0.1161	0.1016
Qinghai	0.0490	0.0482	0.0513	0.0548	0.0533	0.0520	0.0587	0.0591	0.0619	0.0638	0.0552
Ningxia	0.0981	0.0836	0.0794	0.0791	0.0798	0.0808	0.0833	0.0925	0.0991	0.0995	0.0875
Xinjiang	0.1152	0.1192	0.1199	0.1174	0.1186	0.1228	0.1408	0.1379	0.1359	0.1389	0.1267

After measuring and analyzing the ELRC of the four major economic regions and the entire country, the results are presented in Table 4. From a national perspective, China’s regional ELRC has significantly improved during the observation period. The average across the nation increased from 0.1577 in 2012 to 0.2370 in 2021, representing a remarkable growth rate of 50.29%. The highest growth rate was observed between 2017 and 2018. It can be attributed to the establishment of the Emergency Management Department in 2018 and the subsequent release of corresponding plans. The plans emphasized the prioritization of the construction and comprehensive deployment of emergency logistics as a critical project, leading to a rapid enhancement of China’s ELRC. On the regional level, China’s Central, Eastern, Western, and Northeastern regions all have displayed a steady upward trend in ELRC. Among them, the Eastern region exhibited the strongest ELRC, followed by the Central and Northeastern regions, while the Western region displayed a relatively weaker capacity. Both the Eastern and Central regions surpassed the national average in ELRC. However, the Western and Northeastern regions exhibited comparatively lower capacities, falling below the national average. Moreover, from the degree of improvement in ELRC, the Eastern region had an average growth rate as high as 6.12%. The Western region experienced a growth rate of 4.18%, the Central region had a growth rate of 3.41%, and the Northeastern region had a growth rate of 1.86%. This shows that China’s ELRC improvement exhibits significant regional heterogeneity.

**Table 4:** Calculation results of emergency logistics response capacities for different regions in China.

Year	Eastern region	Central region	Western region	Northeastern region	Countrywide
2012	0.1958	0.1819	0.1117	0.1514	0.1577
2013	0.2104	0.1861	0.1185	0.1525	0.1661
2014	0.2209	0.1933	0.1222	0.1532	0.1725
2015	0.2252	0.1892	0.1228	0.1510	0.1730
2016	0.2353	0.1951	0.1262	0.1609	0.1798
2017	0.2528	0.2135	0.1348	0.1702	0.1934
2018	0.2729	0.2284	0.1470	0.1782	0.2083
2019	0.2907	0.2266	0.1479	0.1771	0.2142
2020	0.3133	0.2364	0.1542	0.1747	0.2257
2021	0.3336	0.2448	0.1610	0.1780	0.2370
Mean	0.2551	0.2095	0.1346	0.1647	0.1928

The main reasons for the disparity in regional ELRC in China can be attributed to the following factors: firstly, China’s vast territory, diverse geographical conditions, and complex climate create significant variations in natural conditions among the Western, Eastern, Central, and Northeastern regions. Consequently, these regions face different frequencies, types, and severity levels of public emergencies. Rooted in the frequency of public emergencies in China, the Eastern region experiences the most emergencies, followed by the Central and Western regions, while the Northeastern region faces the least incidents. Due to such differences in public emergency frequency, the accumulated emergency management experience in dealing with the incidents varies significantly across regions. Consequently, the effectiveness of emergency logistics contingency plans formulated by different regions also differs, leading to disparities in their respective ELRC. Furthermore, the lack of an established emergency information data-sharing platform and an incomplete sharing system among relevant departments contribute to *n* inadequate system for allocating emergency supplies across regions. Thus, the Emergency Management Department, the National Food and Strategic Reserves Administration, and other relevant departments are temporarily unable to leverage their respective strengths fully. And then, the limitation hinders the collaborative progress of different regional ELRC. Additionally, there are regional disparities in logistics infrastructure across China. The varying accessibility of emergency logistics transportation routes in different areas leads to differences in their ELRC. Lastly, differences in the scale and quality of disease control centers, the scale of medical resources, and the level of local government investment in emergency logistics collectively result in variations in social emergency security capacity among different regions. As a result, disparities in China’s regional ELRC emerge.

Additionally, further examination of the indicator weights can reveal that despite leading in overall ELRC, certain provinces (e.g., Hainan) in Eastern region exhibit underperformance in “Social Emergency Security Capacity,” primarily due to limited healthcare resources and fiscal expenditure allocation. The Western region showed low scores in “Transport Network Density” and “Optical Fiber Line Length” reflect infrastructure gaps, exacerbated by geographic barriers and sparse population distribution. Besides, it is a fact that moderate “Logistics Carrier Capacity” masks disparities in “Regional Informatization,” where Central region (e.g., Henan) lag in digital connectivity compared to coastal counterparts.

## 5 Analysis of regional heterogeneity in China’s emergency logistics response capacity

In this research, the Dagum Gini coefficient and its subgroup decomposition method were utilized to further analyze the overall heterogeneity of China’s ELRC.

### 5.1 Analysis of overall heterogeneity

The overall heterogeneity can be divided into inter-regional heterogeneity, intra-regional heterogeneity, and hypervariance density. The specific calculation results are presented in Table 5.

**Table 5:** Overall Gini coefficient and decomposition terms of China’s emergency logistics response capacities from 2012 to 2021.

Year	2012	2013	2014	2015	2016	2017	2018	2019	2020	2021
Intra-regional heterogeneity	0.0542	0.0588	0.0593	0.0596	0.0615	0.0629	0.0649	0.0644	0.0650	0.0671
Overall Gini coefficient	0.2347	0.2473	0.2510	0.2540	0.2618	0.2656	0.2696	0.2746	0.2798	0.2875
Inter-regional heterogeneity	0.1262	0.1304	0.1352	0.1391	0.1419	0.1433	0.1422	0.1556	0.1649	0.1701
Hypervariance density	0.0543	0.0581	0.0565	0.0553	0.0584	0.0594	0.0625	0.0545	0.0499	0.0503

Over the observation period, China’s ELRC Gini coefficient grew from 0.2347 in 2012 to 0.2875 in 2021. It indicates a growing trend in the overall heterogeneity of China’s ELRC. From the decomposition terms, both intra-regional heterogeneity and inter-regional heterogeneity demonstrated an increasing trend over the years. However, hypervariance density initially increased and then decreased. Hypervariance density and Intra-regional heterogeneity exhibited smaller values, whereas inter-regional heterogeneity was markedly greater. This phenomenon may be attributed to the distinct differences in economic development and logistics infrastructure across various regions in China. Despite the issuance of emergency plans and relevant policies for emergency logistics in each region, the inter-regional heterogeneity in ELRC continuously increases due to the above differences. And it contributes to further amplifying the overall heterogeneity.

### 5.2 Analysis of intra-regional heterogeneity

Calculating the intra-regional heterogeneity of ELRC in each economic region of China, the results are shown in Table 6. It is evident that from 2012 to 2021, intra-regional heterogeneity within various regions of China exhibited continuous fluctuations. Additionally, in certain years, intra-regional heterogeneity also displayed a cross phenomenon. Specifically, the Eastern region showed the highest level of intra-regional heterogeneity, closely followed by the Western and Northeastern regions. And the Central region exhibited the lowest level of intra-regional heterogeneity. Furthermore, both the Eastern and Western regions demonstrated overall stable intra-regional heterogeneity with a similar upward fluctuation trend, indicating a continuous increasing intra-regional heterogeneity in these regions. The intra-regional heterogeneity within the Central region remained consistently below 0.2, indicating a fluctuating downward trend. Over the period from 2012 to 2021, it declined approximately 0.0273. Conversely, the intra-regional heterogeneity within the Northeastern region exhibited three distinct phases. In the first phase, spanning from 2012 to 2015, there were continuous increases in intra-regional heterogeneity, though none surpassed 0.2. During the second phase, from 2016 to 2018, there were fluctuating increases in intra-regional heterogeneity, all exceeding 0.2. However, the third phase, covering 2019 to 2021, witnessed a trend of diminishing intra-regional heterogeneity, with all values decreasing below 0.2. As a result, compared to other regions, the ELRC in the Northeastern region tends to be more prone to instability.

**Table 6:** Intra-regional heterogeneity of China’s ELRC from 2012 to 2021.

Year	2012	2013	2014	2015	2016	2017	2018	2019	2020	2021
Eastern region	0.1896	0.2258	0.2227	0.2281	0.2359	0.2420	0.2526	0.2468	0.2480	0.2571
Central region	0.1690	0.1408	0.1436	0.1351	0.1372	0.1307	0.1295	0.1421	0.1435	0.1417
Western region	0.1952	0.1988	0.2047	0.1996	0.2055	0.2154	0.2180	0.2105	0.2112	0.2155
Northeastern region	0.1702	0.1789	0.1833	0.1923	0.2085	0.2041	0.2137	0.1978	0.1758	0.1766

The main reason for the changes in these three phases may be that the Northeastern region, comprising the provinces of Liaoning, Heilongjiang and Jilin, serves as a significant industrial base in China. Between 2012 and 2015, China’s market economy’s rapid global development and continuous reforms might have instigated the relocation or shutdown of numerous traditional industrial enterprises, especially in Jilin and Heilongjiang. However, Liaoning province, benefiting from its advantageous coastal location, likely attracted greater foreign investment and the establishment of modern industries. As a derivative of the industrial sector, the logistics sector experienced an expansion in discrepancies as the industrial differences widened. Meanwhile, compared to the coastal logistics of Liaoning, Jilin and Heilongjiang rely more on their relatively underdeveloped railway network. As a result, the ELRC in Liaoning exhibited substantial variation compared to the other two provinces, leading to a persistent rise in intra-regional heterogeneity within the Northeastern region. In 2016–2018, China further propelled its supply-side structural reforms. High-energy-consuming and pollutant-intensive traditional industries faced additional constraints, causing the logistics demand in Jilin and Heilongjiang to contract further. In contrast, modern service sectors and high-tech industries received consistent encouragement and support, with logistics demands in Liaoning experiencing sustained growth. Such shifts might have intensified the disparity between Liaoning and the other two provinces, thereby elevating intra-regional heterogeneity. From 2019 to 2021, the nation actively promoted industrial upgrades and transformations to revitalize the Northeastern region. This signifies that all three Northeastern provinces received new investments and industry inflows, leading to increased logistics demands. Consequently, the ELRC across the provinces improved, indicating a trend toward reduced regional discrepancies.

### 5.3 Analysis of inter-regional heterogeneity

The inter-regional heterogeneity of China’s ELRC was calculated. And the results are presented in Table 7. During the observation period, the highest level of heterogeneity existed between the Western and Eastern regions. However, the lowest level existed between the Northeastern and Central regions. The heterogeneity between other regions appeared between these two extremes, exhibiting a crossover phenomenon. Specifically, from 2012 to 2021, the heterogeneity between the Western and Eastern regions exceeded 0.3, indicating significant disparities between these regions. In opposition, the heterogeneity between the Central and Northeastern regions fluctuated around 0.2, reflecting relatively more minor differences. Likewise, the heterogeneity between the Western and Central regions and between the Northeastern and Western regions showed comparable levels, hovering around 0.25 and 0.22, respectively. Notably, the heterogeneity between the Eastern and other regions exhibited an upward trend over time. This may be directly attributed to the Eastern region’s developed economic conditions and advanced logistics infrastructure. However, it also highlights the evident unevenness in the ELRC across China’s Northeastern, Western, Central, and Eastern regions.

**Table 7:** Inter-regional heterogeneity of China’s emergency logistics response capacity from 2012 to 2021.

Year	2012	2013	2014	2015	2016	2017	2018	2019	2020	2021
Eastern-Central	0.1934	0.2084	0.2096	0.2206	0.2302	0.2257	0.2316	0.2461	0.2550	0.2660
Eastern-Western	0.3152	0.3389	0.3431	0.3491	0.3588	0.3650	0.3649	0.3789	0.3883	0.3976
Eastern-Northeastern	0.2225	0.2609	0.2717	0.2853	0.2867	0.2954	0.3106	0.3260	0.3467	0.3634
Central-Western	0.2677	0.2489	0.2546	0.2415	0.2448	0.2528	0.2484	0.2448	0.2464	0.2450
Central-Northeastern	0.1911	0.1861	0.1967	0.2043	0.2133	0.2123	0.2260	0.2150	0.2135	0.2185
Western-Northeastern	0.2226	0.2203	0.2212	0.2192	0.2367	0.2366	0.2381	0.2235	0.2077	0.2100

### 5.4 Analysis of the sources of regional heterogeneity

**Figure 2:**
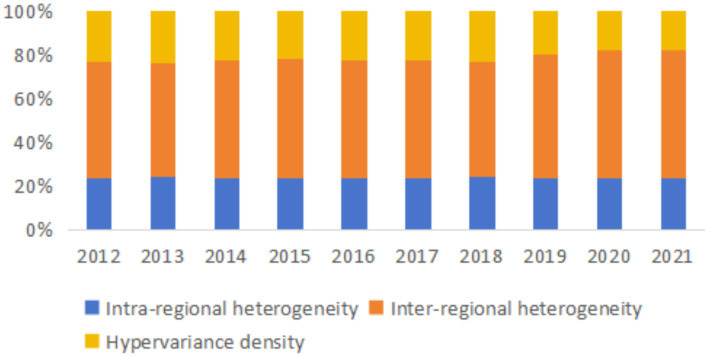
Sources of disparity in China’s emergency logistics response capacity from 2012 to 2021.

The impact levels of the Dagum Gini coefficient’s decomposition terms were calculated. Figure 2 displays the results, with the average contribution rate of inter-regional heterogeneity being the highest, at 55.08%. The average contribution levels of intra-regional heterogeneity is 23.52%. In comparison, the mean contribution rate of hypervariance density is the lowest, at only 21.40%. This shows that the overall heterogeneity in ELRC mainly stems from inter-regional heterogeneity. However, intra-regional heterogeneity and hypervariance density have similar and relatively lower contribution rates, exerting little influence on the overall heterogeneity. The specific reasons may lie in the differences in regional resource endowments and resource allocation. Developed Eastern region enjoys distinct locational advantages and possesses higher levels of economic development and infrastructure. By contrast, other regions lag in various production factors. Consequently, capital investment is more inclined to flow into the Eastern region, significantly boosting its ELRC. Regarding the trends, the contribution rate of hypervariance density fluctuated up and down from 2012 to 2018 but declined from 2019 to 2021. In contrast, both intra-regional and inter-regional heterogeneity contribution rates showed an increasing trend annually.

## 6 Spatiotemporal evolution characteristics of China’s regional emergency logistics response capacity

### 6.1 Dynamic evolution characteristics in the time dimension

This study utilized kernel density estimation to depict the time-based changes in China’s regional ELRC.

#### 6.1.1 Dynamic evolution characteristics at the national level

**Figure 3:**
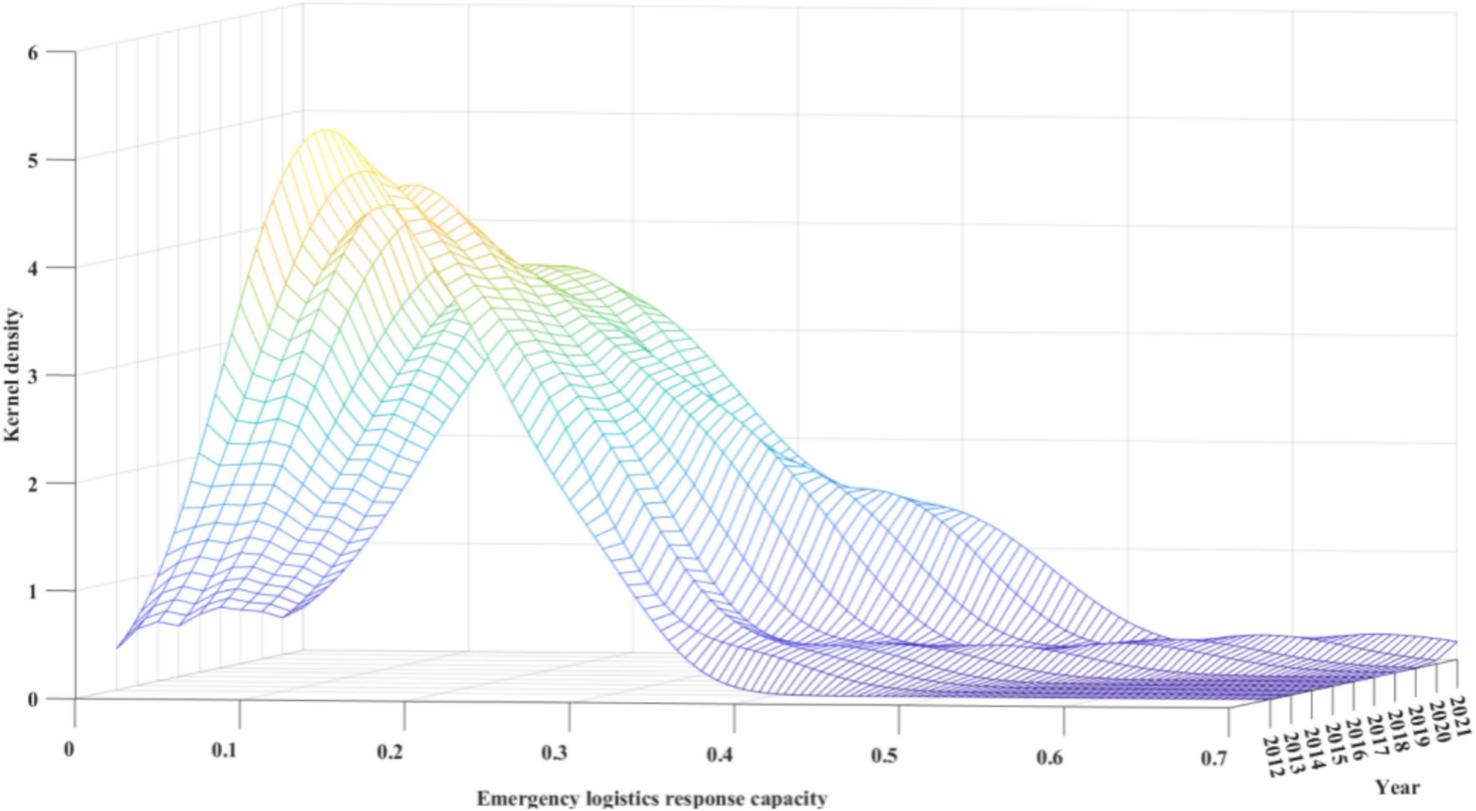
Dynamic evolution of China’s emergency logistics response capacity from 2012 to 2021.

The dynamic evolution characteristics of ELRC under the time dimension at the national level are presented in Figure 3. From observing the distribution positions, the overall centre of the ELRC distribution curve gradually shifted to the right during the observation period, indicating effective improvement in China’s ELRC. Regarding the distribution shape, there was only one peak during the observation period. And the values were mainly concentrated between 0 and 0.2. This seems that China’s ELRC remained relatively stable during the observation period without significant polarization. Additionally, the main peak’s height gradually decreased, and its width became wider. This implies that the measured values of ELRC showed a trend of gradual dispersion during the observation period. In other words, the disparities between different regions’ ELRC have been gradually widening. The stretching of the curve shows that, as the years progressed, the right-tail phenomenon of the kernel density curve decreased. Especially in 2020 and 2021, the kernel density curve showed an upward trend and data aggregation at certain high-level positions. This could be attributed to the widespread outbreak of COVID-19 and the frequent occurrence of severe autumn floods in the middle and lower reaches of the Yellow River, hail disasters in the Eastern region, and snow disasters in the northern part of China. The capability of governments at all levels to respond to and dispatch resources during emergencies has been continually enhanced, subsequently elevating the regional ELRC.

#### 6.1.2 Dynamic evolution characteristics at the economic region level

**Figure 4:**
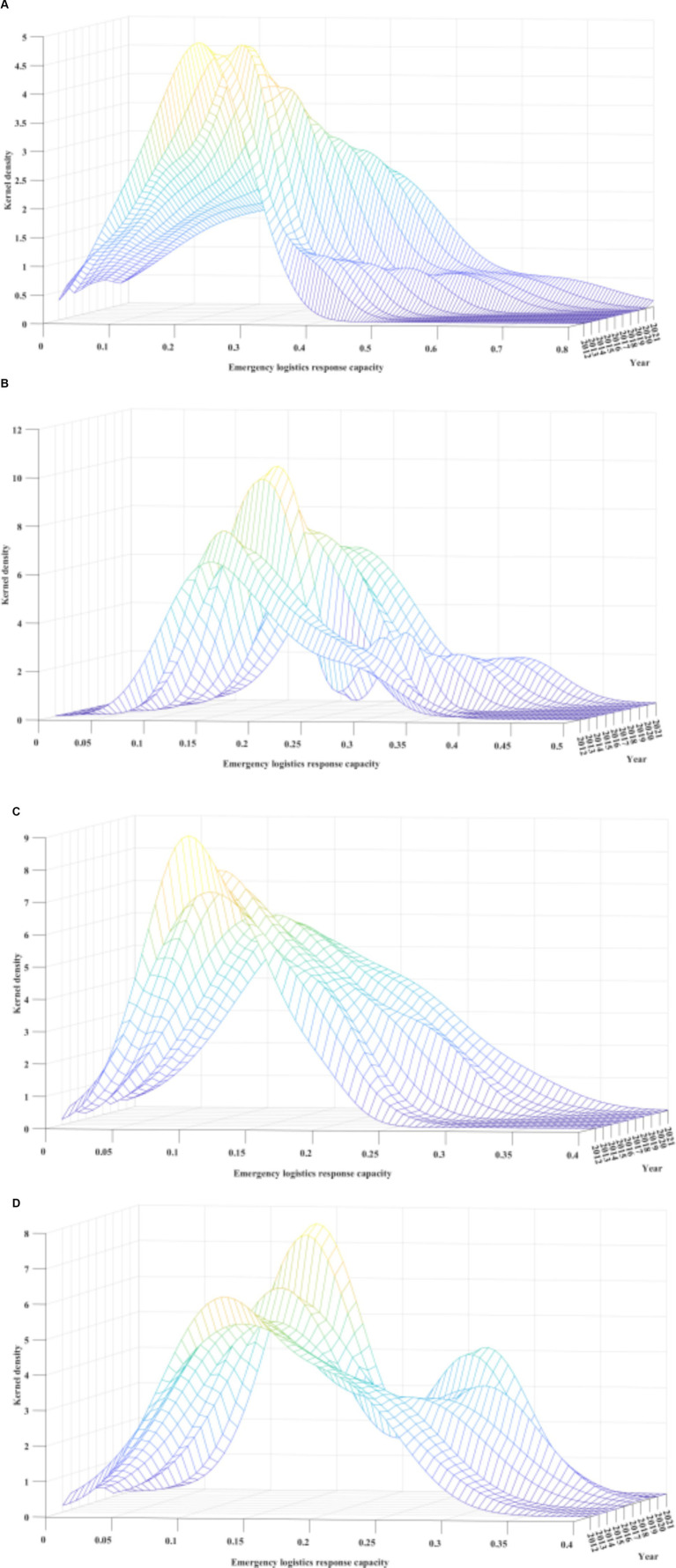
**(A)** Dynamic evolution of emergency logistics response capacity in China’s four major economic regions from 2012 to 2021-Eastern region. **(B)** Dynamic evolution of emergency logistics response capacity in China’s four major economic regions from 2012 to 2021-Central region. **(C)** Dynamic evolution of emergency logistics response capacity in China’s four major economic regions from 2012 to 2021-Western region. **(D)** Dynamic evolution of emergency logistics response capacity in China’s four major economic regions from 2012 to 2021-Northeastern region.

Through the kernel density curve analysis of the ELRC in four major economic regions of China, the results are plotted in Figure 4. The kernel density curves of every region displayed a shift to the right throughout the observation period. This indicates significant improvements in the ELRC of the Eastern, Northeastern, Western, and Central regions. Specifically, the highest ELRC show up in Eastern region, with the peak values of the curve clustering around 0.3. The Central and Northeastern regions followed, with peak values gathering around 0.2 and 0.15, respectively. Lastly, the Western region had the lowest ELRC, with the peak of the kernel density curve ranging between 0.1 and 0.15. Furthermore, both the Western and Eastern regions’ distribution curves showed a gradual decrease in the heights of the main peaks, and their widths became wider. This indicates that the internal differences in ELRC within the Eastern and Western regions have been expanding, which corresponds to the conclusions taken from the Dagum Gini coefficient. Moreover, from the perspective of curve ductility, the right-tail phenomenon of the kernel density curves for the Western and Eastern regions showed a decreasing trend with reduced convergence. This indicates a significant growth trend in these two regions’ ELRC, which is consistent with the analysis presented earlier.

### 6.2 Evolution characteristics in the spatial dimension

In order to better reveal the evolving trends and particular transfer characteristics of China’s ELRC, this paper used the Markov transition probability matrix to analysis. Considering the distinctive features of the research area, the observed ELRC during the sample period was classified into four types using the standard quartile method: poor-level type (I), moderate-level type (II), good-level type (III), and excellent-level type (IV). MATLAB R2021a software was utilized to calculate both the conventional and spatial Markov chain transition probability matrices. The results are presented in Tables 8, 9.

**Table 8:** Traditional Markov chain transition probability matrix of China’s emergency logistics response capacities from 2012 to 2021.

Type	I	II	III	IV	Observation value
I	0.929	0.071	0.000	0.000	70
II	0.000	0.912	0.088	0.000	68
III	0.000	0.000	0.882	0.118	68
IV	0.000	0.000	0.000	1.000	64

**Table 9:** Spatial Markov chain transition probability matrix of China’s emergency logistics response capacities from 2012 to 2021.

Domain type	Type	I	II	III	IV	Observation value
I	I	0.857	0.143	0.000	0.000	7
	II	0.000	0.800	0.200	0.000	5
	III	0.000	0.000	1.000	0.000	5
	IV	0.000	0.000	0.000	1.000	2
II	I	0.949	0.051	0.000	0.000	39
	II	0.000	0.962	0.038	0.000	26
	III	0.000	0.000	0.875	0.125	8
	IV	0.000	0.000	0.000	1.000	9
III	I	0.867	0.133	0.000	0.000	15
	II	0.000	0.923	0.077	0.000	26
	III	0.000	0.000	0.886	0.114	44
	IV	0.000	0.000	0.000	1.000	24
IV	I	1.000	0.000	0.000	0.000	9
	II	0.000	0.818	0.182	0.000	11
	III	0.000	0.000	0.818	0.182	11
	IV	0.000	0.000	0.000	1.000	29

#### 6.2.1 Traditional Markov chain analysis

According to the traditional Markov chain transition probability matrix exhibited in Table 8, from 2012 to 2021, the retention probabilities for the four types of China’s regional ELRC (i.e., poor-level region, moderate-level region, good-level region, and excellent-level region) were 92.9, 91.2, 88.2, and 100%, respectively. These results indicate that China’s regional ELRC maintained stability in its original state during the observation period. Particularly, the excellent-level region had a retention probability of 100%, demonstrating strong stability in its ELRC, consistently maintaining an excellent-level performance. Moreover, the ranking of retention probabilities from highest to lowest is as follows: excellent-level region, poor-level region, moderate-level region, and good-level region. This suggests that China’s overall ELRC is transitioning from a poor-level to a better level, with a trend of continuous improvement. However, the regions with poorer capacity are more likely to remain in their original states, and regions with better capacity tend to maintain their existing ELRC levels. This exhibits specific characteristics of the Matthew effect, likely due to the imperfect logistics infrastructure and emergency event management systems in regions with lower capacity. From the perspective of transition probabilities, there is a 7.1% probability of the ELRC in the poor-level region transitioning to the moderate-level. The moderate-level region has an 8.8% probability of transitioning to the good-level, while the good-level region has an 11.8% probability of transitioning to the excellent-level. It can be observed that the improvement of ELRC occurs only between adjacent levels. And there is no cross-level transition. This appears that the enhancement of China’s ELRC is continuous, and achieving a significant leap in a short period is challenging.

#### 6.2.2 Spatial Markov chain analysis

Based on the traditional Markov chain, this study introduced a spatial weight matrix to incorporate the “spatial lag” factor, thus establishing a spatial Markov chain. By analyzing the spatial Markov chain transition probability matrix presented in Table 9, several inferences can be drawn.

Firstly, the transfer probability matrices differed across various spatial lag types. This indicates that, in the context of heterogeneous ELRC among neighbouring provinces, the probability of ELRC undergoing transfer varies accordingly within this province. It further suggests that spatial factors exert a certain influence on ELRC. Furthermore, the transfer probability matrices exhibit an inclination trend in the upper-right triangular region, revealing a rising pattern of China’s ELRC in the spatial dimension.

Secondly, when the ELRC of the local region is at a moderate-level and neighbouring regions is at a poor-level, the probability of transitioning from the moderate-level to the good-level is 20%. It is significantly higher than the traditional Markov chain’s 8.8%. This suggests that, in the spatial dimension, when adjacent regions have poorer levels, the local region is more inclined to undergo a better-level leap. This might be attributed to the local region’s ability to attract specific emergency logistics resources from neighbouring lower-level areas, thereby elevating its capacity.

Thirdly, when the local regional ELRC reaches an excellent-level, it remains at the excellent-level, irrespective of the level of the adjacent areas. This demonstrates that provinces with excellent ELRC exhibit strong stability. And they are not easily affected by the spatial correlation effects from neighbouring provinces with relatively lower ELRC.

Fourthly, when the ELRC in an adjacent region remains at a poor-level, regions at poor-level, moderate-level, and good-level will exert a more evident absorptive effect on it. For instance, when the ELRC in neighbouring regions is at a poor-level, and the local region also operates at a poor-level, the rate of the local region transitioning from a poor-level to a moderate-level is 14.3%. It significantly exceeds the conventional Markov chain’s probability of 7.1%. This indicates a substantial increase in the likelihood of transitioning from a poor-level region to a moderate-level region. This may be because regions with relatively better or comparable ELRC tend to attract resources from regions operating at a poor-level. Thereby, it exerts positive spatial effects on their own ELRC development. Similarly, when the ELRC in adjacent regions reaches a good-level, the rate of the local region transitioning from a poor-level to a moderate-level is 13.3%. It also far surpasses the conventional Markov chain’s probability of 7.1%. This can be attributed to the implementation of supportive emergency logistics policies by local governments and the directed allocation of resources toward regions with relatively poorer ELRC. These measures act as positive driving forces for enhancing the ELRC of areas operating at a poor-level.

Fifthly, when the ELRC in adjacent regions reaches an excellent-level, the percentage of the local region transitioning from a poor-level to a moderate-level is 0%. It is significantly lower than the traditional Markov chain’s probability of 7.1%. This appears that the excellent-level ELRC in neighbouring regions negatively impacts regions operating at a poor-level, making it challenging for them to achieve rapid improvements in their ELRC. Meanwhile, the percentage of the local region transitioning from a moderate-level to a good-level is 18.2%, higher than the traditional Markov chain’s probability of 8.8%. Similarly, the percentage of the local region advancing from a good-level to an excellent-level is 18.2%, also surpassing the traditional Markov chain’s probability of 11.8%. This reveals that, under the circumstance of adjacent regions with an excellent-level ELRC, regions operating at a moderate-level and a good-level are positively influenced, making it easier for them to undergo transformative upgrades. This could be attributed to the weaker foundations in regions with poor-level ELRC, such as information technology and emergency logistics facilities. Consequently, the radiative driving effect from neighbouring regions with excellent-level capacity is restricted, leading to an increase in spatial disparities in ELRC. In contrast, areas with a certain level of foundation at moderate and good levels are more susceptible to the radiative driving effect from neighbouring regions with excellent-level capacity. This makes it easier for their ELRC to achieve transformative upgrades.

Lastly, even when neighbouring excellent-level regions, the rate of transitioning from a poor-level region to a good-level region remains at 0. And the rate of a transition from a moderate-level region to an excellent-level region also remains at 0. This seems that transitions only occur between adjacent levels of ELRC. A likely explanation is that building emergency logistics infrastructure and enhancing management systems are gradual processes, making significant improvements difficult in a short time. Therefore, such a gradual progression hinders the rapid transformative upgrades of regional ELRC.

## 7 Discussion

This study systematically evaluates the heterogeneity and spatiotemporal evolution of regional ELRC in China through a multidimensional index system and advanced analytical methods. The findings yield critical theoretical and practical implications, while also addressing gaps in existing literature on emergency logistics governance. Below, we synthesize the core insights, contextualize them within broader academic debates, and outline pathways for future research. The central findings can be distilled into three core insights: persistent expansion of regional heterogeneity, gradual progression coupled with stability in spatiotemporal evolution, and dual effects inherent to spatial interdependence.

### 7.1 Key contributions to theory and practice

First, our analysis reveals a persistent and widening spatial disparity in ELRC across China, characterized by a pronounced “East–West gradient.” The Dagum Gini coefficient decomposition demonstrates that inter-regional heterogeneity (contributing 55.08% to overall inequality) dominates this pattern, particularly between the economically advanced Eastern region and underdeveloped Western provinces. This aligns with the “core-periphery” theory in regional economics (78), where resource concentration in core areas exacerbates spatial imbalances.

Second, unlike prior studies focusing on static evaluations (27, 29), our spatiotemporal approach uncovers the self-reinforcing nature of regional disparities: high-capacity regions (e.g., Guangdong) exhibit strong stability (100% retention probability), while low-capacity regions (e.g., Qinghai) face structural barriers to improvement, reflecting a “Matthew effect” in emergency resource allocation.

Third, the study identifies dual spatial spillover effects shaping ELRC evolution. Spatial Markov chain analysis highlights that proximity to high-capacity regions can both facilitate and hinder local capacity development. For instance, moderate-capacity regions adjacent to high-capacity neighbors show an 18.2% probability of upgrading (vs. 8.8% in isolation), suggesting technology diffusion and policy emulation. Conversely, low-capacity regions bordering high-capacity areas experience suppressed upgrading probabilities (0% for low to moderate transitions), likely due to resource siphon effects. This duality echoes debates on “trickle-down” versus “polarization” effects in regional development (79), offering empirical evidence specific to emergency logistics systems.

### 7.2 Heterogeneity and influencing factors

The heterogeneity in China’s regional ELRC is not only a reflection of spatial disparities but also a product of multifaceted influencing factors. Our analysis reveals that resource endowments, governance capacity, and spatial spillovers collectively shape the uneven distribution of ELRC across regions.

First, this study conjects that the resource endowment gap between the Eastern and Western regions is a primary driver of heterogeneity. The Eastern region benefits from advanced logistics infrastructure (e.g., high-grade highways, dense railway networks) and robust digital connectivity (e.g., fiber-optic coverage), which are critical for rapid disaster response. In contrast, the Western region’s rugged terrain and underdeveloped transportation networks hinder the efficient allocation of emergency resources. This aligns with the findings of Chen et al. (13), who highlighted the role of geographical constraints in exacerbating regional imbalances in logistics development.

Second, governance capacity plays a pivotal role in shaping ELRC. Provinces with higher fiscal expenditure on transportation and healthcare (e.g., Guangdong, Jiangsu) demonstrate stronger emergency preparedness and response capabilities. This is consistent with the literature on disaster management (75), which emphasizes the importance of institutional frameworks in enhancing resilience. However, our study extends this perspective by revealing that policy implementation gaps—such as uneven enforcement of emergency plans and limited interdepartmental coordination—further amplify regional disparities.

Finally, socioeconomic factors such as population density, industrial structure, and climate risks also contribute to heterogeneity. For example, the Eastern region’s high population density and exposure to frequent disasters (e.g., typhoons, floods) have driven investments in resilient infrastructure and emergency management systems. In contrast, the Northeastern region’s reliance on traditional industries and aging population has limited its capacity to adapt to emerging challenges. These findings echo the work of Zhou et al. (2), who identified socioeconomic vulnerabilities as key determinants of disaster resilience.

### 7.3 Limitations and future research directions

While providing novel insights, this study has limitations that warrant further exploration:

1. Due to time difficulties and constraints in data acquisition, the 2012–2021 dataset predates recent mega-disasters (e.g., 2023 Henan floods), limiting insights into post-pandemic resilience dynamics. Future work should incorporate real-time disaster response data to validate the index system’s predictive validity.
2. It has not delved into the exploration of influencing factors on ELRC, especially the role of factors such as resource endowments, geographical landscapes, logistics infrastructure, and climate. Therefore, future research can consider incorporating the study and analysis of the influencing mechanisms on regional ELRC to provide theoretical support and decision-making references for enhancing ELRC.
3. China’s centralized governance model may limit the transferability of findings to decentralized systems. Comparative studies across political regimes (e.g., U.S. federal vs. EU supranational models) could identify context-specific optimization strategies.

Future research should prioritize two avenues:

1. Integrating Geographic Information Systems (GIS) and machine learning algorithms to analyze the impacts of dynamic factors—such as climate change and population mobility—on ELRC. For example, simulate resource flows under stochastic disaster scenarios to stress-test regional coordination protocols. Or, embed climate risk projections into ELRC assessments to address escalating environmental uncertainties.
2. Explore synergies between emergency logistics networks and sustainable development goals (SDGs), particularly SDG 9 (infrastructure) and SDG 11 (resilient cities).

## 8 Conclusions and implications

### 8.1 Research conclusions

The research constructed a comprehensive index system for regional ELRC and applied the entropy-weighted TOPSIS method to measure the ELRC of 30 provincial-level administrative regions in China from 2012 to 2021. Subsequently, the Dagum Gini coefficient, its subgroup decomposition, Markov chain and kernel density estimation, analysis were used to conduct heterogeneity and spatiotemporal evolution analyzes. The conclusions achieved are as follows.

From 2012 to 2021, the ELRC of various provinces in China have shown significant improvement. Particularly, provinces with better ELRC are mainly concentrated in the Eastern regions. In contrast, provinces in the western regions exhibit relatively poorer levels of ELRC.

The overall Gini coefficient for China’s ELRC presents a continuous expansion trend, and the heterogeneity of ELRC is mainly derived from inter-regional heterogeneity. Moreover, the ELRC in the Central, Eastern, Western, and Northeastern regions have been steadily improved. No polarization phenomenon was observed, and the performance appeared relatively stable. However, the regional disparities have gradually expanded over time. The analysis using the Markov chain reveals that China’s ELRC is undergoing a gradual transition from a poorer level to a better level, showing a continuous upward trend. However, at the same time, it exhibits specific characteristics of the Matthew effect.

### 8.2 Policy implications

Drawing from the research conclusions above, this paper offers the following policy recommendations:

The first is targeted infrastructure investment. For the Western region, prioritize high-grade highway construction (e.g., Sichuan-Qinghai corridors) and subsidize rural optical fiber deployment to bridge digital divides. Modernize railway freight networks in Northeastern region and incentivize public-private partnerships to upgrade aging transport fleets. Fully harness market resources, refine the mainline transportation and regional distribution systems of emergency logistics and augment the ELRC to various unexpected incidents.

The second is inter-regional collaborative mechanism development. Establish East–West Resource Redistribution Platforms, such as leveraging Guangdong’s advanced ELRC to mentor western provinces (e.g., Qinghai) by sharing emergency reserves and conducting joint training programs. Besides, construct a unified data-sharing platform for the visualization, dissemination, and coordinated command of emergency logistics information. Especially in the Central region, develop cross-provincial data hubs (e.g., Wuhan-Xi’an) to enhance real-time information sharing during multi-hazard events.

The third is dynamic performance monitoring framework. Implement a tiered evaluation system to track ELRC advancements. Provinces achieving tier transitions (e.g., low-to-medium) should receive fiscal incentives, while underperforming regions must be mandated to develop corrective action plans. Concurrently, instate inter-regional technological exchange and collaboration platforms, promoting the sharing and dissemination of best practices. Encourage enterprises and institutions in the Eastern region to foster technological collaboration and exchanges with their Western counterparts. Furthermore, the government should incentivize logistics enterprises to invest and expand in the Western region through tax incentives or fiscal subsidies.

## Data Availability

Publicly available datasets were analyzed in this study. This data can be found here: “China Statistical Yearbook” and the website of National Bureau of Statistics (www.stats.gov.cn).

## Footnotes

^1^Multi-Granularity Linguistic Assessment (MGLA) is a method used in decision-making processes that involves evaluating and synthesizing information based on linguistic terms of different granularity levels, allowing for more nuanced and flexible analysis of subjective data.

^2^ www.stats.gov.cn
